# A Multi-Faceted Approach to Analyse the Effects of Environmental Variables on Geographic Range and Genetic Structure of a Perennial Psammophilous Geophyte: The Case of the Sea Daffodil *Pancratium maritimum* L. in the Mediterranean Basin

**DOI:** 10.1371/journal.pone.0164816

**Published:** 2016-10-17

**Authors:** Olga De Castro, Antonietta Di Maio, Mirko Di Febbraro, Gennaro Imparato, Michele Innangi, Errol Véla, Bruno Menale

**Affiliations:** 1 Department of Biology, University of Naples Federico II, Naples, Italy; 2 Department of Biosciences and Territory, University of Molise, Pesche (Isernia), Italy; 3 Department of Electrical and Information Technology Engineering, University of Naples Federico II, Naples, Italy; 4 Department of Environmental, Biological, Pharmaceutical Sciences and Technologies, Second University of Naples, Caserta, Italy; 5 Research unit Botany and Modelling of Plant Architecture and Vegetation, University of Montpellier, Montpellier, France; Università di Pisa, ITALY

## Abstract

The Mediterranean coastline is a dynamic and complex system which owes its complexity to its past and present vicissitudes, e.g. complex tectonic history, climatic fluctuations, and prolonged coexistence with human activities. A plant species that is widespread in this habitat is the sea daffodil, *Pancratium maritimum* (Amaryllidaceae), which is a perennial clonal geophyte of the coastal sands of the Mediterranean and neighbouring areas, well adapted to the stressful conditions of sand dune environments. In this study, an integrated approach was used, combining genetic and environmental data with a niche modelling approach, aimed to investigate: (1) the effect of climate change on the geographic range of this species at different times {past (last inter-glacial, LIG; and last glacial maximum, LGM), present (CURR), near-future (FUT)} and (2) the possible influence of environmental variables on the genetic structure of this species in the current period. The genetic results show that 48 sea daffodil populations (867 specimens) display a good genetic diversity in which the marginal populations (i.e. Atlantic Sea populations) present lower values. Recent genetic signature of bottleneck was detected in few populations (8%). The molecular variation was higher within the populations (77%) and two genetic pools were well represented. Comparing the different climatic simulations in time, the global range of this plant increased, and a further extension is foreseen in the near future thanks to projections on the climate of areas currently—more temperate, where our model suggested a forecast for a climate more similar to the Mediterranean coast. A significant positive correlation was observed between the genetic distance and Precipitation of Coldest Quarter variable in current periods. Our analyses support the hypothesis that geomorphology of the Mediterranean coasts, sea currents, and climate have played significant roles in shaping the current genetic structure of the sea daffodil especially during LGM because of strong variation in coastline caused by glaciations.

## Introduction

The Mediterranean coastline is a dynamic and complex system which owes its complexity to its past and present vicissitudes, e.g. complex tectonic history, climatic fluctuations, and prolonged coexistence with human activities [1]. All of these causes have contributed to the simultaneous presence of various environments, along with the occurrence of species whose geographic distributions reflect different past events [2, 3, 4]. In detail, the Mediterranean Sea and its coastlines have experienced changes in their configuration due to climate change also in the recent past (i.e. glacial period), which determined extreme changes in marine levels [2, 5]. In addition, Mediterranean coasts are harsh habitats, characterized for example by direct exposure to sea breeze and continuous salt spray, low nutrient and fresh water availability, strong radiation, and high temperatures [6]. In spite of many studies aimed to understand the effects of environmental changes of the recent past (late-middle Pleistocene, Quaternary) and current period, to date no information is present on the effects of differential contribution of environmental variables on the genetic structure of psammophilous species from the Mediterranean coasts. A plant species that is widespread in these habitats is the sea daffodil, *Pancratium maritimum* L. (Amaryllidaceae), which is a perennial clonal geophyte of coastal sands (fixed/mobile sand dunes and beaches) of the Mediterranean and neighbouring areas, well adapted to the stressful conditions of sand dune environments [6, 7, 8].

Because of the buffering effects of its life history traits (e.g. great longevity, overlapping generations, and long juvenile phase) on changes in genetic structure, *P*. *maritimum* offers greater advantages over short-lived organisms for exploring how much genetic variation is associated with current and/or past/future environmental conditions using statistical simulations [9, 10], as already documented in *Asplenium fontanum* (L.) Bernh. [11], *Eucalyptus gomphocephala* DC. [12] and *Taxus baccata* L. [13].

Briefly, *P*. *maritimum* is a bulbous species capable of vegetative reproduction by bulbils [14]; flowers are herkogamous and leaves appear during different seasons (hysteranthous) [15]; seeds are dispersed by water and wind due to their specialized structure [16, 17]; no consensus is available on the breeding system (self-compatible vs. incompatible) [14, 18, 19, 20, 21, 22], and no hybrids with other species of *Pancratium* in nature are known; its haploid number is *n* = 11 [23]. Little is known on the longevity of the sea daffodil, although potted plants at Naples and Catania Botanical Gardens have a documented age of > 80 years. For further details about the species, see Materials and Methods (Study Species).

In this study, an integrated approach was used, combining genetic and environmental data with a niche modelling approach, aimed to investigate: (1) the effect of climate change on the geographic range of this species at different times {past: the last interglacial (LIG, ~120–140 kya BP), the last glacial maximum (LGM, ~21 kya BP); the present conditions (CURR, ~1950–2000); and near-future (FUT, ~2070)} and (2) the possible influence of environmental variables on the genetic structure of this species in the current period. As a side issue, a simulation analysis was performed correlating current genetic structure with past environmental data (LIG and LGM periods), to evaluate as past climatic changes may have influenced present-day genetic structure of this plant.

To this aim, we employed nuclear microsatellite markers (nrSSR, Short Sequence Repeat) to investigate patterns of the diversity and genetic structure in *P*. *maritimum* on a large and representative sample of populations and individuals. This choice is motived by the nature of SSRs which are codominant, neutral, highly polymorphic and reproducible markers. To evaluate a distribution model for this plant, the World Climate Database was consulted, using the available climatic information for three historical periods (LIG, LGM, CURR, and FUT).

## Materials and Methods

### Study species

*Pancratium maritimum* belongs to genus *Pancratium* L., which likely evolved during the Miocene [23]. No information is available at present about the time of origin of the species. During summer, the plant produces white, scented flowers (2–14), the mean number of ovules per flower is c. 56.6 [24], and each flower produces c. 10–20 seeds. The long seed longevity of this species is well documented, as reported in Mira et al. [25]. The seeds are black and extremely light (c. 4–8 mg) and are specialized for both anemochory and hydrochory [16, 17, 26]. Salinity and water stress do not fatally affect seed viability or germination over a moderate term [26, 27]. Medrano et al. [19] suggested that the breeding system of the sea daffodil varies among populations according to ecological conditions (e.g. ecologically marginal or geographically peripheral populations). The above mentioned authors performed a study in a south-western Spanish population in which *P*. *maritimum* was found to be a self-compatible and autogamous species, in contrast to the data of Eisikowitch and Galil [18], who studied a *Pancratium* population from Israel, in which the plants were found to be self-incompatible and exclusively pollinated from hawk moths.

This plant is currently endangered along the Mediterranean coasts due to the loss of its natural habitat caused by human pressure (i.e. sunbathing, excess flower sampling, and sand dune erosion) [28, 29, 30, 31, 32, 33]. To date, only three interesting but localized geographical studies are available on the population genetic of this species, resulting in a partial understanding of its conservation status: Zahreddine et al. [14], who used dominant markers (RAPD) on populations from Lebanon; Grassi et al. [20], who employed dominant markers (AFLP) for northern Italy; and Sanaa and Fadhel [21], who used isozymes (codominant markers) on plants from Tunisia.

Nevertheless, this species has not yet been evaluated by the IUCN (International Union for Conservation of Nature, Red List, http://www.iucnredlist.org/apps/redlist/search), leading to concerns regarding the future of this species [14, 20, 21, 34]. A noteworthy example of the severity of the risk for this species is its total disappearance from the island of Ischia (Naples, southern Italy) because of human impact [35].

### Ethics statement

The sampling has been performed without destroying the populations of *P*. *maritimum*. A small portion of leaf tissue was sampled from each individuals and stored to -80°C freezer nearby Dept. Biology (Naples, Italy). No specific permissions were required for the sampling in all locations (Table 1) because the plant is not endangered according to IUCN; sampling, however, was not destructive as reported above.

**Table 1:** Sampled localities, human pressure values and genetic characteristics of the 48 *Pancratium maritimum* populations that were included in the genetic analyses.

Country	Code	Locality	Latitude	Longitude	HFP	N	P_PL_	P_AN_	A_T_	A_R_	S	H_O_	H_E_ (uH_E_)	F_IS_
Algeria	AE	Annaba	36.9067	8.1292	28	18	100	0	39	3.85	1	0.72	0.70 (0.72)	-0.004
Algeria	AG	Bousfer (Oran)	35.7511	-0.8294	53	20	100	0.8	23	2.66	0	0.49	0.54 (0.56)	0.116
Algeria	AL	Mazafran (Zeralda)	36.6987	2.8036	68.3	22	100	0	22	2.61	1	0.45	0.49 (0.5)	0.113
Croatia	CA	Korcula Island	42.9151	17.0831	29.2	23	100	2.9	35	3.47	6	0.51	0.66 (0.67)	0.244*
Croatia	IV	Vis Island	43.0582	16.2522	28	8	100	0	16	2.57	0	0.75	0.57 (0.61)	-0.248
France	CJ	Ajaccio (Corsica)	41.9347	8.6239	17.8	24	100	1.4	37	3.39	2	0.71	0.63 (0.64)	-0.103
France	CO	San-Nicolao (Corsica)	42.3742	9.5324	59	24	67	2.7	21	2.42	0	0.52	0.41 (0.41)	-0.262
France	CC	Porto Vecchio (Corsica)	41.6706	9.3754	32.8	24	100	0	34	3.50	0	0.67	0.66 (0.68)	0.019
France	CS^†^	Propiano (Corsica)	41.6743	8.8930	53	24	100	0	21	2.72	0	0.51	0.59 (0.6)	0.154
France	M	Marseillan	43.3239	3.5631	61.5	20	83	2.5	19	2.21	0	0.45	0.39 (0.4)	-0.122
Greece	AT	Kalamaki (Crete)	35.0278	24.7600	46.5	20	100	0	25	2.67	0	0.87	0.56 (0.57)	-0.535
Greece	F	Chersonissos (Crete)	35.2953	25.4825	62.4	20	100	0	35	3.43	1	0.73	0.66 (0.68)	-0.077
Greece	GA	Kalogria	38.1612	21.3656	37	22	100	0	26	2.87	0	0.63	0.55 (0.56)	-0.128
Greece	H	Rhodes Island	36.4532	28.2175	50	10	83	2.5	20	2.72	5	0.65	0.49 (0.51)	-0.286
Israel	K	Herzliya	32.1704	34.7995	62	19	100	5.3	34	3.64	6	0.55	0.64 (0.66)	0.175
Italy	VV	Albarella (Venice)	45.2765	12.3033	37	6	83	0	15	2.36	0	0.69	0.46 (0.5)	-0.429
Italy	VN	Ca' Roman (Venice)	45.2388	12.2939	39.4	6	83	0	14	2.32	0	0.67	0.48 (0.52)	-0.311
Italy	CP	Calambrone (Pisa)	43.5996	10.2936	78	24	100	0	25	2.72	0	0.58	0.51 (0.52)	-0.114
Italy	I	Cuma (Naples)	40.8485	14.0475	76	24	100	4.1	42	3.68	5	0.62	0.64 (0.66)	0.059
Italy	ER	Eraclea (Venice)	45.5485	12.7720	46	5	100	0	17	2.60	0	0.57	0.46 (0.51)	-0.133
Italy	N	Gallipoli (Lecce)	40.0826	18.0093	84	24	100	0	26	3.12	1	0.78	0.63 (0.64)	-0.217
Italy	Z^†^	Le Cesine (Lecce)	40.3556	18.3451	27.6	11	83	0	19	2.66	0	0.68	0.52 (0.54)	-0.271
Italy	CT	Marina di Camerota (Salerno)	39.9974	15.3706	54	24	100	2.1	22	2.62	0	0.67	0.52 (0.53)	-0.279
Italy	A	Marina di Palidoro (Rome)	41.9137	12.1437	79	22	83	0	19	2.51	0	0.59	0.46 (0.47)	-0.269
Italy	P	Marina di Pietrapaola (Cosenza)	39.5445	16.8611	52.1	20	100	0	29	3.23	1	0.64	0.61 (0.62)	-0.032
Italy	T	Marina di Sibari (Cosenza)	39.7467	16.4950	52.9	19	100	0.85	30	3.21	0	0.57	0.56 (0.58)	0.022
Italy	PS	Pineto (Teramo)	42.5849	14.0894	70.3	20	100	1.7	28	2.74	5	0.49	0.46 (0.47)	-0.047
Italy	G	Vieste (Foggia)	41.8438	16.1806	36.7	20	83	0	24	2.80	0	0.62	0.49 (0.51)	-0.224
Italy	B	Arzachena (Sardinia)	41.0892	9.5617	48	23	100	0.72	34	3.35	1	0.59	0.58 (0.6)	0.013
Italy	SA	Oristano (Sardinia)	40.0436	8.3972	58.7	24	100	0	35	3.21	1	0.56	0.58 (0.59)	0.045
Italy	SR	Pula (Sardinia)	39.0095	9.0231	59	18	100	0	19	2.32	0	0.52	0.44 (0.45)	-0.149
Italy	D	Villasimius (Sardinia)	39.1237	9.5254	48	19	100	0.9	23	2.46	0	0.57	0.46 (0.48)	-0.200
Italy	FS	Foce Simeto (Sicily)	37.3991	15.0896	56	14	100	0	22	2.80	0	0.67	0.58 (0.6)	-0.117
Italy	E	Selinunte (Sicily)	37.5814	12.8029	48	20	100	0.83	24	2.79	0	0.73	0.55 (0.56)	-0.319
Libya	L	Damah	32.7733	21.3514	34	16	100	0	26	3.22	1	0.71	0.61 (0.63)	-0.131
Malta	O	Rampla tat-Torri (Mellieha)	35.9915	14.3617	n.d.	20	100	0.85	28	3.08	2	0.71	0.57 (0.58)	-0.215
Morocco	MB^†^	Bouknadel (Salé)	34.1492	-6.7367	69	11	83	0	16	2.45	0	0.52	0.47 (0.49)	-0.046
Morocco	MA	Mehdya (Kenitra)	34.2570	-6.6794	58.3	5	83	0	14	2.26	0	0.63	0.43 (0.47)	-0.394
Morocco	Q	Mohammedia	33.7000	-7.4149	82	18	100	0	19	2.49	0	0.50	0.49 (0.5)	0.004
Portugal	J^†^	Fonte da Telha (Almada)	38.6560	-9.2498	57.7	11	83	0	18	2.50	0	0.58	0.48 (0.5)	-0.155
Spain	BE	Minorca (Balearic Islands)	40.0530	4.0559	34	24	83	0	21	2.54	0	0.62	0.47 (0.48)	-0.303
Spain	R	Almeria	36.8293	-2.4558	62	24	100	0	33	3.51	2	0.49	0.66 (0.67)	0.282*
Spain	W	Cangas de Morrazo (Pontevedra)	42.4209	-8.6734	96.8	24	83	0	19	2.26	0	0.38	0.4 (0.41)	0.075
Spain	S	El Portil (Huelva)	37.2086	-7.0500	62.4	20	100	8.3	26	3.05	0	0.55	0.57 (0.58)	0.063
Spain	KA	L'Ampolla (Tarragona)	40.8011	0.6996	86	5	83	0	15	2.45	0	0.47	0.45 (0.5)	0.067
Spain	SM	Malaga	36.7197	-4.4044	94.7	20	100	0.83	23	2.80	0	0.49	0.54 (0.55)	0.121
Tunisia	TM	La Marsa	36.8868	10.3311	99	22	100	1.5	29	3.48	0	0.62	0.69 (0.7)	0.124
Turkey	KR	Karasu (Sakarya)	41.1048	30.7092	58.4	6	83	2	13	2.14	1	0.50	0.44 (0.48)	-0.059
	**Mean ± SE**				56 ± 2.9	18 ± 0.91	94.44 ± 1.26	0.89 ± 0.23	25 ± 1.05	2.84 ± 0.06	0.88 ± 0.24	0.6 ± 0.021	0.54 (0.56) ± 0.012 (0.013)	-0.09 ± 0.03

HPF, Human Footprint value (%); N, number of individuals sampled per population; P_PL_, polymorphic loci (%); P_AN_, null allele (%); A_T_, total number of alleles observed across all loci; A_R_, allelic richness; S, total number of private alleles observed; H_O_, observed heterozygosity; H_E_, expected heterozygosity (uH_E_, unbiased, expected heterozygosity); F_IS_, inbreeding coefficient;

^†^, evidence of bottleneck under TPM and SMM (one-tailed P-values of excess of gene diversity) considering population with n > 10 (α = 0.05).

Asterisk marks significant departure (P < 0.05) from Hardy–Weinberg equilibrium following sequential Holm-Bonferroni correction. n.d. no datum.

### Plant sampling for genetic analyses

**Fig 1:**
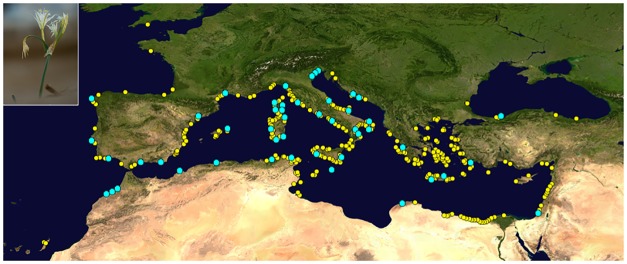
Map of 48 *Pancratium maritimum* populations used in the genetic analyses (blue circles) and 537 accessions used in the Species Distribution Models (SDMs) (yellow + blue circles) (map by NASA Earth Observatory).

A total of 867 mature individuals were sampled from 48 localities, covering most of the distribution area of *P*. *maritimum* and representing as much diversity in ecological conditions as possible (Table 1 and Fig 1). Field collections were conducted from 2009 to 2013. The number of individuals that were sampled per population ranged from 5 to 24 (Table 1). To avoid sampling one genetic individual more than once because of vegetative reproduction through bulbil production, each sampled plant (clump) was usually separated from the next by at least 10 m.

The sampled *P*. *maritimum* populations were geo-referenced using a geographic information system (GIS) and overlapped on the ortho-photo of Google Earth for successive analyses. To infer how the present human impact affects the 48 sampled *P*. *maritimum* populations, we calculated the Human Footprint Value (HFP) through the Global Human Footprint Database, v2 (1995 -2004) [36] using a circular buffer of a 1-km ray around each of the 48 sampled *P*. *maritimum* populations (Table 1). The Euclidean geographic distance matrix among the populations was calculated posing mainland as an insurmountable barrier (nautical distance) using the package “gdistance” in the R environment software [37] (S1 File). This choice was made because of the presence of the hydrochory as an important dispersal phenomenon in the sea daffodil biology.

### Genetic analysis

By using the microsatellite library obtained from *P*. *maritimum* [37], after a preliminary amplification test, six nuclear microsatellites (SSRs) were chosen (SSR15, SSR25, SSR27, SSR30, SSR31, and SSR38) and genotyped for the 48 populations in study (867 individuals). The genomic DNA extraction, PCR conditions, and genotyping screening were as previously described in De Castro et al. [23] and Di Maio and De Castro [38]. Standard procedures were used to minimize scoring errors as suggested in DeWoody et al. [39].

To achieve genotyping accuracy, we analysed SSR data with the Micro-Checker version 2.2.3 software [40] to determine the existence of stuttering (slight changes that occur in the allele size during PCR), dropout alleles (large alleles do not amplify as efficiently as small alleles), and null alleles [39]. For each microsatellite locus, we assessed genetic polymorphism by calculating the total number of alleles (A_T_), observed and expected heterozygosity (H_O_ and H_E_), and inbreeding indexes (F_IS_) using GenAlEx version 6.5 software [41]. The fixation index (F_ST_) was computed with FreeNA software, which implements the ENA correction method to amend for the positive bias that is induced by the presence of possible null alleles in F_ST_ estimation and provides an accurate estimation of F_ST_ in the presence of null alleles [42]. Deviation from Hardy-Weinberg equilibrium (HWE) and linkage disequilibrium (LD) between microsatellites were tested using GENEPOP, version 4.1 software [43], within each population and for each locus. Sequential Holm-Bonferroni correction was used to determine the significance levels for all tests with an initial level of 0.05 [44] using the macro Holm-Bonferroni Sequential Correction: An EXCEL Calculator—version 1.2 [45].

For each population, we estimated genetic diversity across all loci using the percentage of polymorphic loci (P), observed number of alleles (A_T_), number of private alleles (S), average observed (H_O_), expected heterozygosity (H_E_), and unbiased heterozygosity (uH_E_), the latter representing the expected heterozygosity under HWE within populations, adjusted for sample size using GenAlEx version 6.5 software. The allelic richness (A_R_) was also calculated for each population using the FSTAT version 2.9.3.2 software [46]. Briefly, A_R_ is the number of alleles independent of sample-size as determined using the rarefaction method of El Mousadik and Petit [47]. The inbreeding coefficient F_IS_ was calculated using the same previous software [48], and deviation from zero (Hardy–Weinberg genotypic proportions) was also evaluated by permuting alleles within populations (5000 permutations) using sequential Holm-Bonferroni correction [45].

The pairwise genetic differentiation F_ST_ was estimated with FreeNA software corrected for null alleles [42] using 1000 bootstraps to compute 95% confidence intervals. The presence of isolation-by-distance (IBD) was tested by correlating Rousset’s [49] genetic distance (F_ST_⁄1-F_ST_) and geographical distance (logarithmic scale) between populations using a Mantel test in Arlequin version 3.1 software [50] with 1000 permutations.

An analysis of molecular variance (AMOVA) was used to partition the total genetic variation within and among population components using Arlequin version 3.5.1.3 software. The significance of the AMOVA was assessed with 1000 permutations of the data.

To estimate the distribution of individuals among natural genetic groups (*K*), the dataset was also analysed by Bayesian-model-based clustering methods, as implemented in the software STRUCTURE version 2.3.4 software [51, 52, 53, 54]. The analysis was performed without prior information concerning the geographic origin of the accessions. The STRUCTURE algorithm was run using the ‘‘admixture model”, assuming a ‘‘correlation among allele frequencies”, with 30 independent replicate runs per K value (number of clusters) ranging from 1 to 10. Each run involved a burning period of 50,000 iterations and a post burning simulation length of 100,000 iterations. To obtain the appropriate K from the data according to Evanno et al. [55], we used the STRUCTURE HARVESTER version 0.6.93 software [56]. Finally, the estimated cluster membership coefficient (Q) matrices of multiple runs that were generated by STRUCTURE were analysed using CLUMPP version. 1.1.2 software [57] to calculate the average pairwise similarity (H’). To graphically represent the results obtained, the average results of the assignments of STRUCTURE were plotted on maps generated with ArcMap as implemented in ArcGIS version 10.3 software (ESRI, Redlands, CA, USA).

Finally, a demographic analysis was performed using the BOTTLENECK version 1.2.02 software [58]. Of the several available tests that are implemented in the software (i.e. sign test [59], including standardized differences test [60], Wilcoxon sign-rank test [61], and model-shift tests [62]), only the Wilcoxon sign-rank was chosen because it is the most accurate in cases of a low number of polymorphic loci (SSRs loci < 20) and individuals per population (n > 10) [58, 61]. Briefly, a population that has been subjected to a drastic reduction in the number of individuals shows a reduction in the number of alleles (k) and heterozygosity. In particular, the number of alleles is reduced more than the decrease of heterozygosity, with the result that, in the population, the observed heterozygosity is higher than expected based on the number of alleles (k), assuming a constant population size [60]. A Wilcoxon sign-rank test was performed to evaluate whether the difference across loci significantly differed from zero. Using this method, the bottleneck signature can be detected up to two and four Ne (effective population size) generations after the event [60]. Because Wilcoxon sign-rank test as performed in BOTTLENECK was not robust, with less than 10 individuals for population [58], we analysed only those populations with n > 10. A two-phase mutation model (TPM) was used for the Wilcoxon sign-rank test. This model combines the infinite alleles model (IAM) and the single-step mutation model (SSM) [63] and produces more appropriate results with a small number of loci [58]. We ran the TPM simulation as 90% one-step mutations and 10% multistep changes under 10,000 permutations. A comparative analysis was also performed with the SSM model as suggested by Piry et al. [58]. The probability of significant excess heterozygosity over all loci (P) was determined using a one-tailed Wilcoxon sign-rank test.

### Species Distribution Models

To evaluate current, past and future presence suitability for *P*. *maritimum*, we trained Species Distribution Models (SDMs) using environmental predictors referring to the current time, and then projecting the models over past and future versions of these predictors. Occurrence data used for the training of SDMs were gathered from our database, literature, public databases and personal communication with experts (S2 File and Fig 1). We filtered the data by removing duplicated records and those with unrealistic coordinates. In addition, the spatial accuracy of the records was assessed by including only occurrence data points given to at least 2 decimal places [64], obtaining a final dataset of 537 occurrences (S2 File and Fig 1). A set of 10,000 pseudoabsences were randomly placed over a region identified by all the WWF Terrestrial Ecoregions [65] that included species records [66, 67].

As an initial set of environmental predictors, we considered the 19 bioclimatic variables derived from the WORLDCLIM database at a spatial resolution of 30 arc-seconds (ca. 1 km) [68] and the Euclidean distance from the coastline. To take into account the pairwise correlation between the predictors, the variables were subselected considering a variance inflation factor (VIF) less or equal to 3 [69]. The final environmental predictors used for the model training were: Euclidean distance from the coastline, Temperature Seasonality (BIO4), Mean Temperature of Wettest Quarter (BIO8), Mean Temperature of Driest Quarter (BIO9), Precipitation Seasonality (BIO15), and Precipitation of Coldest Quarter (BIO19). All the procedures were carried out with the packages “spatstat”, “maptools”, “rgeos” and “raster”, in the R environment software [37]. Models were projected over present-day, past and future environmental conditions. Model projections over past climates were carried out for the last glacial maximum (LGM; ~26–20 kya BP) {two models: the Community Climate System Model (CCSM) and the Model for Interdisciplinary Research on Climate (MIROC} and the last inter-glacial (LIG; ~120–140 kya BP) [70, 71]. For the model projections over the future climates, we considered the future climate model outputs for 2070, made available through the Intergovernmental Panel on Climate Change (IPCC) Data Distribution Centre (http://ipcc-ddc.cru.uea.ac.uk). In particular, we used the climate change model output “HadGEM2” from the V assessment report [72], for the less and most impacting IPCC’s climate scenarios: RCP2.6 and RCP8.5. These scenarios describe possible future trends in concentration of greenhouse gases (GHG), with RCP2.6 forecasting emissions to reach a peak around 2010–2020, then decline substantially, and RCP8.5 a continue emissions’ rise throughout the 21st century [72]. For computational reasons, all the models were projected at a resolution of 2.5 arc-minutes (ca. 5 km) within a distance of 50 km from the coastline. Projections were made over a geographic area centred on the Mediterranean basin and ranging between 20° and 55° parallels (S2 File and Fig 1). The potential species distributions were predicted using an ensemble forecasting approach, as implemented in the R package “biomod2” [73]. We considered the following six modelling algorithms: generalized linear models (GLM), generalized additive models (GAM), generalized boosted models (GBM), random forests (RF), multivariate adaptive regression spline (MARS) and maximum entropy models (MAXENT), covering all the main modelling classes implemented in biomod2 (for further details, see [73]). The occurrence dataset was randomly split into a 70% sample, used for the calibration of the model, and a remaining 30%, used to evaluate model predictive performance, repeating the procedure 10 times and averaging the results. The predictive performance of each model was assessed by measuring the area under the receiver operating characteristic curve (AUC) [74] and the true skill statistic (TSS) [75]. To avoid using poorly calibrated models, only projections from models with AUC ≥ 0.7 and TSS ≥ 0.4 were considered in all subsequent analyses [76, 77]. For the current, past and future predictions we averaged the model projections to obtain final consensus maps The model averaging was performed by weighting the individual model projections respectively by their AUC score and averaging the results, as this method was shown to be particularly robust [78]. Finally, model projections were reclassified into presence and absence using a threshold that maximizes sensitivity (the percentage of presence correctly predicted) and specificity (the percentage of absence correctly predicted) [79]. Such a threshold is one of the most accurate according to [80, 81, 82, 83, 84, 85].

### Impact of environmental variables on genetic data

To determine the contribution of present environments on genetic differentiation, we tested for the relationship between the pairwise F_ST_ and environmental distance while controlling for geographic distance, as in Mayol et al. [13]. This choice was made because it is possible to have incorrect correlations between F_ST_ and environmental distance (i.e. isolation by adaptation) due to strong patterns of IBD; thus, it is possible to discern the contribution of geographic and environmental distance in the obtained pattern.

We computed Euclidean distance matrices based on the values of the environmental variables that were used for the model training and measured at the locations of the 48 *P*. *maritimum* populations that were included in the genetic analyses (Table 1, Fig 1, S1 File). Considering that all of the 48 populations occur within 1 km of the coastline, this predictor did not incorporate a source of environmental variability among the populations and was excluded from the calculation of the environmental distance matrices (hereafter, “climatic distance matrices”). We used geographic distance matrices among the populations as described in the previous paragraph (Plant Sampling for genetic analyses). Tests were performed using partial Mantel correlations [86] and multiple matrix regressions (MMRR; [87]) within the R environment [37]. Significance tests were based on 10,000 permutations. To reduce the risk of spurious correlations, particularly for less conservative MMRR tests, we only considered those correlations that were significant with both methods [13]. The same procedures were applied to investigate the contribution of past climate to the current genetic differentiation (F_ST_). To investigate the correlation of past climate and pairwise F_ST_, we retained only those populations in which a suitable environment existed for *P*. *maritimum* persistence during the LGM and LIG periods. Accordingly, we selected those occurrence points among the 48 populations occurring in cells that were predicted to be suitable by the SDMs during the LGM and LIG periods [13].

## Results

### Genetic analyses

A Micro-Checker analysis provided no evidence of scoring errors due to large allele drop-out or stutter peaks in our final dataset. A presence of null alleles was detected in 19 populations (39.6%, Table 1): higher frequency was observed in a Spanish population (S, P_AL_ = 8.3%), in Israel (K, P_AL_ = 5.3%), and in a southern Italian population (I, P_AL_ = 4.1%). The remaining populations presented a range of null alleles from 0.8%-2.9% (Table 1). The number of alleles that were detected per locus ranged from 16 to 27 (mean 22 ± 1.53); the H_O_ among all of the loci was 0.6 (± 0.12), with a range of 0.29–0.98; the H_E_ ranged from 0.42 to 0.65 with an average of 0.54 (± 0.05); the F_IS_ value across all of the loci was -0.08 (± 0.14), ranging from -0.56 to 0.36; and the F_ST_ values ranged from 0.14–0.33 (mean 0.24 ± 0.03) and 0.14–0.32 (mean 0.23 ± 0.03) with ENA correction (Table 2).

**Table 2:** Genetic parameters for each of the six nuclear microsatellite loci that were used in this study of *Pancratium maritimum*.

Locus	Allele size range	A_T_	H_O_	H_E_	F_IS_	F_ST (no ENA)_	F_ST (ENA)_
**SSR15**	207–241	24	0.44	0.43	-0.02	0.33	0.33
**SSR25**	191–250	27	0.98	0.63	-0.56	0.2	0.2
**SSR27**	206–235	22	0.38	0.42	0.09	0.25	0.24
**SSR30**	113–215	16	0.92	0.64	-0.44	0.14	0.14
**SSR31**	119–171	20	0.29	0.45	0.36	0.31	0.28
**SSR38**	126–156	23	0.58	0.65	0.12	0.21	0.20
**Mean ± SE**		22 ± 1.53	0.60 ± 0.02	0.54 ± 0.01	-0.08 ± 0.14	0.24 ± 0.03	0.23 ± 0.03

A_T_, total number of observed alleles; H_O_, observed heterozygosity; H_E_, expected heterozygosity; F_IS_, inbreeding in individuals relative to their population; F_ST_, inbreeding with not and ENA correction.

There was no evidence of linkage disequilibrium between the 720 SSR locus-by-locus tests across the 48 populations, except for seven combinations (⩽ 0.97%) (pop. AT, loci 27–38; pop. I, loci 25–30 and 30–31; pop. A, loci 15–38; pop. Q, loci 27–31; pop. W, loci 25–27; and pop. TM, loci 25–27), using sequential Holm-Bonferroni corrections. Of the 288 population-by-locus tests, 115 (39.9%) deviated significantly from HWE after sequential Holm-Bonferroni correction. However, these tests were not limited to a single locus or sampling site; thus, all six of the loci were retained for further analysis.

**Fig 2:**
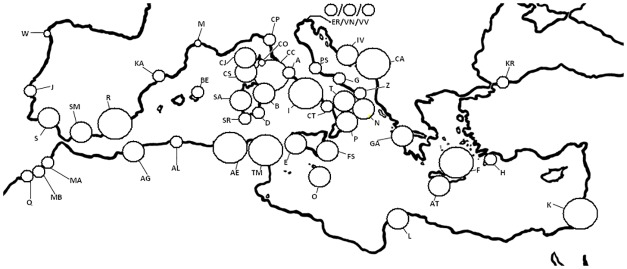
Distribution of genetic diversity (uH_E_, unbiased expected heterozygosity in HWE) in the 48 *Pancratium maritimum* populations as analysed with genetic data (867 individuals). The values for uH_E_ are indicated by the circle size gradient {○(0.72−0.66)>○(0.64−0.55)>○(0.54−0.45)>○(0.44−0.4)}.

The genetic diversity parameters that were analysed for the 48 *P*. *maritimum* populations are shown in Table 1 and Fig 2. Briefly, the level of polymorphic loci (P_PL_) within populations ranged from 67 to 100%. Private alleles (S) were found in 17 out of 48 populations. For each population, the values for H_O_ ranged from 0.38 to 0.87 (mean 0.6 ± 0.02), those of H_E_ ranged from 0.39 to 0.7 (mean 0.54 ± 0.01), and similar values were obtained with sample size correction (uH_E_), as shown in Table 1. The allelic richness (A_R_) ranged from 2.14 to 3.85 (mean 2.84 ± 0.06). The F_IS_ values ranged from -0.55 (AT, Crete) to 0.28 (R, Spain) with an average of -0.09 (± 0.03), and very low levels of inbreeding were detected in the majority of the analysed populations, except for ten populations with F_IS_ values greater than 0.1, as shown in Table 1 {AG, Algeria—Bousfer (0.12); AL, Algeria—Mazafran (0.14); CS, Corsica—Propiano (0.19); CA, Croatia–Korkula Island (0.24); K, Israel–Herzliya (0.19); R, Spain—Almeria (0.28); W, Spain–Cangas de Morrazo (0.1); KA, Sapin,—L’Ampolla (0.11); SM, Spain—Malaga (0.17); and TM–La Marsa (0.11)}. Deviation from Hardy–Weinberg genotypic proportions was significant in two of these populations (CA, Croatia–Korkula Island and R, Spain—Almeria) after sequential Holm-Bonferroni correction (Table 1).

Similar pairwise F_ST_ values were obtained when correcting or not for the presence of null alleles (ENA correction). The corrected values ranged from 0.046 (AL, Algeria—Mazafran; CC, Corsica–Porto Vecchio) to 0.53 (H, Rhodes Island; W, Spain, Pontevedra), with an average of 0.23 (S1 File). According to AMOVA, 23% of the total genetic variation could be attributed to differences among populations and 77% to differences among individuals within populations. The correlation between genetic and geographic distances was positive (r = 0.28, P < 0.001), indicating the existence of a probable isolation-by-distance pattern. Similar results were also obtained using the pairwise F_ST_ not correcting for null alleles (data not shown).

**Fig 3:**
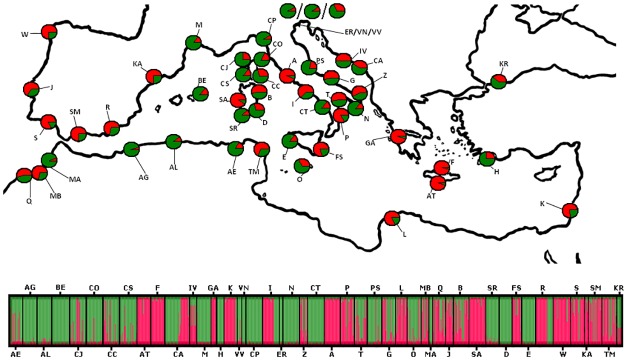
Genetic clusters (K) obtained for the 48 populations (867 individuals) of *Pancratium maritimum* populations using STRUCTURE (K = 2). Different colours (green and red) indicate different genetic clusters.

The assignment of accessions into groups or gene pools was further assessed based on the assignment tests that were carried out with STRUCTURE. These analyses identified a major peak at K = 2 and another peak at K = 4 (S3 File). The highest average pairwise similarity (H’) was associated with K = 2. Even if this Bayesian method identified an optimal partition in two genetic pools (red and green), an unclear geographical pattern was observed (Fig 3): both genetic pools were always observed in each population with different frequencies. Both of the gene pools presented a similar frequency (green = 53.23% and red = 46.77%), although the red genetic pool was more frequently represented in eastern and western peripherical populations (Fig 3).

According to the changes in population size, the Wilcoxon sign-rank test revealed a recent demographic bottleneck in four populations under both the TPM and the SSM model (Table 1): CS, Corsica–Propiano (P_TPM_ = 0.008; P_SSM_ = 0.023); Z, southern Italy–Le Cesine (P_TPM_ = 0.031; P_SSM_ = 0.023); MB, Morocco–Bouknadel (P_TPM_ = 0.031; P_SSM_ = 0.031); and J, Portugal–Fonte da Telha (P_TPM_ = 0.016, P_SSM_ = 0.031). Seven populations were discarded by the Wilcoxon sign-rank test due to a low number of individuals (n < 10, Table 1)**:** IV, Croatia—Vis Island; VV, Italy–Albarella (Venice); VN, Italy, Ca’ Roman (Venice); ER, Italy–Eraclea (Venice); MA, Morocco–Mehdya; KA, Spain–L’Ampolla; and KR, Turkey–Karasu.

### Species Distribution Models

**Fig 4:**
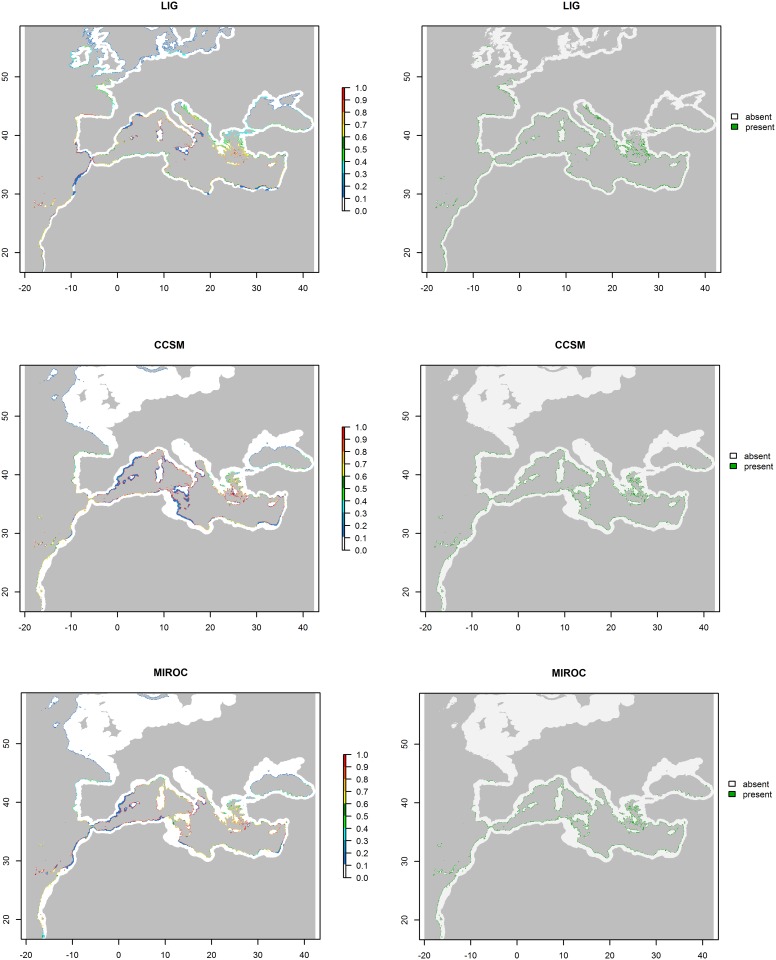
Potential distribution of *Pancratium maritimum* depicted as the probability of presence (a) and presence/absence (b) during two time periods: last interglacial (LIG, ~120,000–140,000 yr BP) and last glacial maximum (LGM-CCSM and LGM-MIROC, ~21,000 yr BP) using two different paleoclimate layers: the Community Climate System Model (CCSM) and the Model for Interdisciplinary Research on Climate (MIROC). In panel “a”, the suitability values range from 0 (white) to 1 (red). In panel (b), the green pixels represent cells of predicted presence, whereas the white pixels refer to cells of predicted absence. Each cell has an area of ca. 25 km^2^.

**Fig 5:**
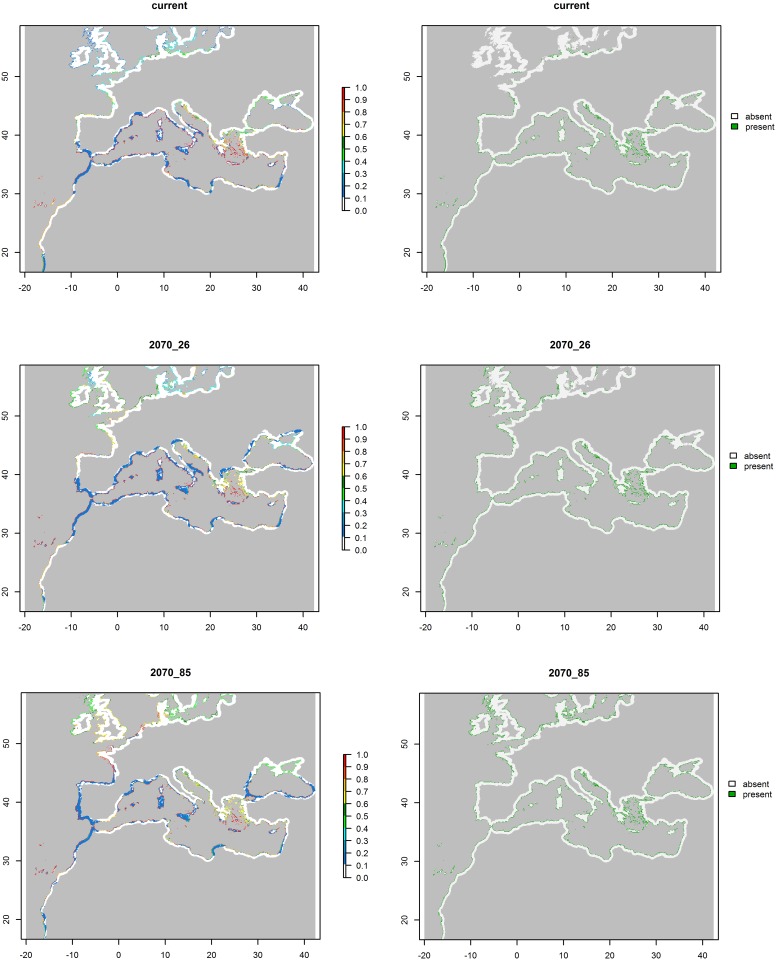
Potential distribution of *Pancratium maritimum* depicted as the probability of presence (a) and presence/absence (b) during two time periods: current period (CURR, 1950–2000) and near-future conditions (FUT-2070) assuming different rates of global CO_2_ increase (RCP2.6 and RCP8.5). In panel “a”, the suitability values range from 0 (white) to 1 (red). In panel (b), the green pixels represent cells of predicted presence, whereas the white pixels refer to cells of predicted absence. Each cell has an area of ca. 25 km^2^.

SDMs reached high predictive performance, with a mean testing AUC of 0.987 (SD = 0.013) and a mean TSS of 0.952 (SD = 0.040). Both AUC and TSS values are ranked as “excellent” according to the classifications proposed by Swets [77] and Landis and Koch [76], respectively. In Figs 4 and 5 and S2 File are shown the different potential distributions in the different periods and the relative contribution to the models and related response curves of each environmental predictor, respectively.

**Fig 6:**
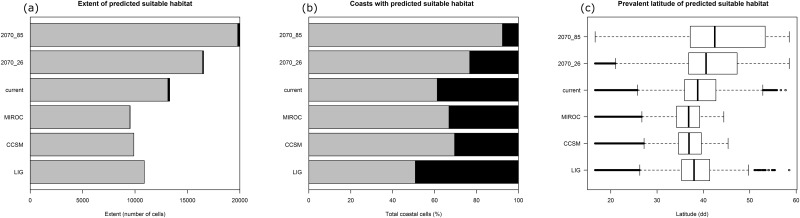
Statistics of the current, past and future potential distribution of *Pancratium maritimum*. Bars in the panel (a) refer to the extent of the predicted distributions in each time interval, also depicting the fraction of suitable cells directly contiguous (grey bars) or not (black bars) to the sea. Panel (b) depicts the percentage of the coasts in the projection area, predicted as suitable (grey bars) or not (black bars), for the species in each time interval. Box plots in panel (c) report the latitude values of the predicted suitable cells in each time interval. The black, thick lines refer to the median, the boxes to the interquartile range and the whiskers to ± 1.58 multiplied by the interquartile range and divided by the square root of the number of values.

The current potential distribution of *P*. *maritimum* was predicted to occupy the coastline of the entire Mediterranean basin, also including the southern and north-western coasts of the Black Sea (Fig 4). In addition, suitable areas were predicted along the eastern coast of the Atlantic Ocean, ranging from northern Africa to France. The species reduces its predicted suitability northward, with just a small portion of suitable cells in southern United Kingdom and along the coasts of the North Sea (Fig 4). For the current time, more than 99% of the suitable cells were predicted as directly contiguous to the sea (Fig 6a), and almost 60% of the total coastlines in the projection area resulted suitable for the species (Fig 6b). When projected over past environments, species potential distribution resulted less extended than the current one, especially the predictions for LGM (Fig 5), with all the suitable cells occurring in adjacency with the sea (Fig 6a), and occupying approximately 50% and 70% of the total coastlines in LIG and LGM, respectively (Fig 6b). Similarly, the species was predicted to occur in a narrower latitudinal range in the past with respect of the current one, with the northern margins of the distribution resulting maximally limited during LGM (Fig 6c). When projected to 2070, the species potential distribution was predicted to widen its extent under both climate change scenarios (Fig 4), with the most impacting one (RCP8.5) showing the highest increase (Fig 6a). Although the percentage of future suitable cells occurring in adjacency with the sea remained almost identical to the current time (i.e. more than 99% of the total suitable cells), *P*. *maritimum* was predicted to extend its southern and northern margins, especially under RCP8.5 climate change scenario (Fig 6c). In accordance with this predicted increase in 2070, the species potential distribution resulted to occupy from 76% to 92% of the total coasts in the projection area, depending on the climate change scenario (Fig 6b).

### Impact of environmental variables on genetic data

For the current time, Mantel partial correlations and MMRR revealed a significant positive association between the pairwise F_ST_ and Precipitation of Coldest Quarter (BIO19) (r_Clim_ *=* 0.427, b_Clim_ = 0.392, P < 0.001), whereas correlations with other predictors were not significant (Table 3). SDMs projections onto past climates suggest that suitable conditions would have existed for the persistence of 2 and 42 populations of *P*. *maritimum* during the LGM and LIG, respectively. During the LIG, six current localities were not present {northern Italy—Adriatic sea side (VV, VR, ER), south-eastern Corsica (CS), and southern and northern Spain (KA and W)}; in contrast, during the LGM, only AG (northern Africa, Algeria) and D (south-eastern Sardinia) localities were present. Thus, we restricted the calculation of correlations with pairwise F_ST_ to only LIG predictions. LIG climates contributed similarly as did the current climates to genetic divergence, with a positive association between the pairwise F_ST_ and Precipitation of Coldest Quarter (BIO19) (r_Clim_ = 0.331, b_Clim_ = 0.304, P < 0.001) and with all others predictors yielding non-significant relationships. Congruent results were also obtained using the pairwise F_ST_ not correcting for null alleles (data not shown).

**Table 3:** Multiple matrix regression (MMRR) coefficients (b) and Partial Mantel (PM) correlation (r) between genetic distance (F_ST_) and environmental variables for the current time (CURR, 1950–2000).

CURRENT TIME (CURR, 1950–2000)			
	MMRR		PM
Predictors	b_Geo_	b_Clim_	r_Clim_
F_ST_–BIO4/Geo	0.2847**	0.0588ns	0.0536ns
F_ST_–BIO8/Geo	0.3165***	0.098ns	0.1099ns
F_ST_–BIO9/Geo	0.31***	0.0379ns	0.0417ns
F_ST_–BIO15/Geo	0.2961***	0.0604ns	0.0625ns
F_ST_–BIO19/Geo	0.2229***	0.3917***	0.4276***

BIO4, Temperature Seasonality; BIO8, Mean Temperature of Wettest Quarter; BIO9, Mean Temperature of Driest Quarter; BIO15, Precipitation Seasonality; BIO19, Precipitation of Coldest Quarter.

***, P < 0.001;

**, P < 0.01;

*, P < 0.05;

ns, not significant

Positive significant tests for both MMRR and PM tests are in bold.

## Discussion

Our integrated analysis supports the hypothesis that the environmental variables and the sea currents as well as the Mediterranean coasts have played important roles in shaping the genetic structure of *P*. *maritimum* in the course of time. According to our genetic data (Table 1, Fig 2), only the 8% of populations show genetic signatures of recent bottleneck, whereas the majority of the investigated sea daffodil populations show a good genetic diversity (uH_E_ = 0.56 ± 0.013), although not uniformly distributed (F_ST_ = 0.23 ± 0.03); marginal populations (i.e. Atlantic Sea populations), in fact, present a lower genetic diversity, as already observed in literature [88, 89]. The observed genetic diversity is likely linked to the biology of *P*. *maritimum*, as, for example, the presence of a bulb, which allows survival under difficult conditions. It is important to note in this respect that genetic diversity is not correlated with human pressure in any of the analysed populations (Table 1). Even if small scale studies are still required, this lack of correlation may be explained by the fact that, even if the (beautiful) flower are over-collected [90], bulbs are not usually eradicated. In addition, the frequent asexual reproduction and large seed production with high germination rates and long juvenile (> 4 years to flowering) and mature phase determine both a good genetic turnover and preservation along time. Analysed populations show infrequent inbreeding (F_IS_ = 0.09 ± 0.03) and this is related to the different reproductive strategies used by *P*. *maritimum* according to different external pressures (i.e. vegetative reproduction and out/in-crossing pollinations). Molecular variation is higher within (77%) than among populations; genetic differentiation among populations (see pairwise F_ST_ in S1 File) is quite variable as a consequence of our sampling strategy (i.e. large geographical distance among populations); and two gene pools are present among the populations in this study (red vs green pool, Fig 3), supporting the hypothesis that geomorphology of the Mediterranean coasts and sea currents have played significant roles in shaping the genetic structure of the sea daffodil. In fact, the two gene pools are mixed without a very clear phylogeographical structure; this may depend upon several factors, including the clone-longevity of *P*. *maritimum* and its specialized seeds, which use two different dispersion modalities, sea currents and wind, that, in combination, can boost the diffusion of this plant. Similar patterns were observed in another coastal species plant, *Calystegia soldanella* (L.) R.Br., which presents a similar biology (i.e. perennial and clonal plant, great seed longevity, and sea-water dispersal) [91]. In the aforementioned study, the authors postulate that the lack of geographic genetic structure of AFLP molecular markers is caused by long-distance seed dispersal and the great clone longevity of the plant. According to literature, sea currents can constitute both a barrier and directional transport routes in the seed dispersion of *P*. *maritimum* [92, 93], and the complexity of sea current circulations in the Mediterranean can determine different patterns in coastal plants in which a general model of distribution pattern is not easily applied (e.g. *Cakile maritima* Scop., *Eryngium maritimum* L., *Salsola kali* L., *Halimione portulacoides* (L.) Aellen, *Crithmum maritimum* L. in [94]; *Carex extensa* Gooden. in [95] and references therein).

Comparing the different climatic simulations in time (Figs 4–6), the global range of *P*. *maritimum* increased, and a peak is foreseen in the near future thanks both to the fact that areas currently with more temperate climate should reach a climate more similar to that of the present-day Mediterranean coast and also to the good capacities of resistance, resilience and adaptability of *P*. *maritimum* [6, 8, 33, 96]. For example, this species has faced several climate changes during recent past (e.g. glacial and interglacial cycle). During these periods, the sand dune coasts were subject to strong geomorphological changes, as observed in the LGM period, where the sea levels significantly decreased [97]. This was also confirmed by our paleo-distribution modelling for the LGM, when only two of the present-time populations (northern Africa and south-eastern Sardinia) were inferred as physically present, due to a strong variation in coastline caused by glaciations [98]. Indeed, the Mediterranean Sea level decreased to approximately -140 m below its present position [97], and the north Adriatic Sea became part of an extensive alluvial plain [99, 100]. However, it is beyond dispute that *P*. *maritimum* has recolonized the sand dune habitat in time, as indirectly documented by our current genetic data and modelling simulations.

In addition, the species distribution models do not suggest that the distribution of the sea daffodil is linked to distinct climatic regimes, even if the bioclimatic variables have great importance for the life cycle of *P*. *maritimum* (see environmental predictors in Materials and Methods, S2 File and Fig 6). This result is also in accordance with the results of a partial Mantel tests and MMRRs, showing significant positive present-day correlation at present between the genetic distance and Precipitation of Coldest Quarter variable (Table 3). Similar correlation patterns were observed in the past, confirming the importance of this variable (see results). The importance of rainfall as a selective agent in *Pancratium* spp. is already documented by Holdsworth [101] and Perrone et al. [6]. According to Holdsworth [101], rainfall, but not temperature shocks, induced anthesis in two African *Pancratium* species (*P*. *trianthum* Herb. and *P*. *hirtum* A.Chev.) living in a dry environment (e.g. savannah). According to Holdsworth [101], even if the development of inflorescence initials in the bulb occurs throughout the year, the author showed that the subsequent emergence and flowering were determined by the abundant rainfall. In fact, in perennial geophytes with hysteranthous leaves like the sea daffodil, an accumulation of storage materials is a prerequisite for flowering, as indicated by Burtt [102]. Dafni et al. [15] reported that the abundance of flowering in *P*. *maritimum* is influenced to a limited extent by the current climatic conditions, and even after the accumulation of the initial critical mass, flowering can occur almost every year. In this case, the presence of a rainy period must be one of the prerequisites for the improved fitness of the sea daffodil as supposed in Holdsworth [101] and Dafni et al. [15]. Considering the available information and our genetic/climatic correlations, it is likely that storage materials in *P*. *maritimum* is directly proportional to winter rainfall [102], with a positive correlation with flowers production as well [15, 101]. Thus, an increase of precipitation may cause an increment in the number of flowers which may be pollinated, producing new genetic turnover which may increase the genetic diversity in time and in a favourable climatic context.

We refrain from speculations on the possible future scenarios emerging by our model; however, it is now certain that the increased temperature, as amply demonstrated in literature [103, 104], significantly affects the coastlines (e.g. inundation and erosion) [31, 105, 106, 107], modifying sea daffodil habitats. The extent of these modifications is debatable. In fact, a less pessimistic scenario (RCP2.6) assumes sustained net negative GHG emissions after year 2070, with global mean temperature projected to rise in an interval between 0.3 and 1.7°C by the late-21st century (2081–2100 average) and a global mean sea level projected to rise by 0.26 to 0.55 m; in contrast, the RCP8.5 pathway, which assumes continued anthropogenic GHG emissions, with a global warming of 2.6 to 4.8°C and an increased sea level of 0.45 to 0.82 m or the same time period [72, 108, 109, 110].

## Conclusions

Genetic data of the sea daffodil were combined with past and present climatic information to assess the role of the environment in the observed patterns of *P*. *maritimum* genetic structure. Thanks to this multi-faceted approach, we highlight the importance of the inclusion of complementary, non-genetic data, for better interpreting genetic patterns, and in order to have a better understanding of the evolution of plants in space and time. This integrated approach may be used in other organisms to create a complete and informative database, providing new tools to explore the effect of climate factors on the patterns of genetic diversity in a wider context.

## Supporting Information

S1 File Pairwise matrices of 48 *Pancratium maritimum* populations. Sheet 1: Pairwise geographic (nautical) distance. Sheet 2: Pairwise geographic (nautical) distance (log scale). Sheet 3: Pairwise distance matrix F_ST_ with ENA correction. Sheet 4: Pairwise distance matrix F_ST_ with no ENA correction. Sheet 5: Pairwise distance matrix F_ST_/1-F_ST_ with ENA correction. Sheet 6: Pairwise distance matrix F_ST_/1-F_ST_ with no ENA correction.
(XLS)

S2 File Species Distribution Models inputs/output. Sheet 1: Dataset of the *Pancratium maritimum* accessions that were used in both the Species Distribution Models (SDMs) and the genetic analyses. Sheet 2: Relative contribution to the models and related response curves of each environmental predictor.
(XLS)

S3 File Structure output. Graph of delta K values to determine the ideal number of groups that were present in the accessions of *Pancratium maritimum* and genetic clusters (K) as obtained for the 48 *P*. *maritimum* populations (867 individuals) using STRUCTURE (K = 4).
(DOCX)

## References

[1] Stanley DJ, Wezel F-C. Geological Evolution of the Mediterranean Basin. New York: Springer; 1985.

[2] Biju-Duval B, Letouzey J, Montadert L. Structure and evolution of the Mediterranean basins. DSDP Initial Reports XLII, 951–984; 1977.

[3] Thompson JD. Plant evolution in the Mediterranean. Oxford: Oxford University Press; 2005.

[4] Lionello P. The Climate of the Mediterranean Region From the Past to the Future. London: Elsevier; 2012.

[5] Goffredo S, Dubinsky Z. The Mediterranean Sea Its history and present challenges. London: Springer; 2014.

[6] Perrone R, Salmeri C, Brullo S, Colombo P, De Castro O. What do leaf anatomy and micro-morphology tell us about the psammophilous *Pancratium maritimum* L. (Amaryllidaceae) in response to sand dune conditions? Flora. 2015; 213: 20–31.

[7] Khedr AHA, Abbas MA, Wahid AAA, Quick WP, Abogadallah GM. Proline induces the expression of salt-stress-responsive proteins and may improve the adaptation of *Pancratium maritimum* L. to salt-stress. J Exp Bot. 2003; 54: 2553–2562. 10.1093/jxb/erg277 14512386

[8] Camprubi A, Abril M, Estaun V, Calvet C. Contribution of arbuscural mycorrhizal symbiosis to the survival of psammophilic plants after sea water flooding. Plant Soil. 2012; 351: 97–105.

[9] Austerlitz F, Mariette S, Machon N, Gouyon P-H, Godelle B. Effects of colonization processes on genetic diversity: differences between annual plants and tree species. Genetics. 2000; 154: 1309–1321. 10757772 10.1093/genetics/154.3.1309 PMC1461003

[10] de Witte LC, Stöcklin J. Longevity of clonal plants: why it matters and how to measure it. An Bot. 2010; 106: 859–870. 10.1093/aob/mcq191 PMC2990663 20880935

[11] Bystriakova N, Ansell SW, Russell SJ, Grundmann M, Vogel JC, Schneider H. Present, past and future of the European rock fern *Asplenium fontanum*: combining distribution modelling and population genetics to study the effect of climate change on geographic range and genetic diversity. Ann Bot. 2014; 113: 453–465. 10.1093/aob/mct274 24284816 PMC3906967

[12] Nevill PG, Bradbury D, Williams A, Tomlinson S, Krauss SL. Genetic and palaeo-climatic evidence for widespread persistence of the coastal tree species *Eucalyptus gomphocephala* (Myrtaceae) during the Last Glacial Maximum. Ann Bot. 2014; 113: 55–67. 10.1093/aob/mct253 24284819 PMC3864724

[13] Mayol M, Riba M, González-Martínez SC, Bagnoli F, de Beaulieu JL, Berganzo E, et al Adapting through glacial cycles: insights from a long-lived tree (*Taxus baccata*). New Phytol. 2015; 208: 973–986. 10.1111/nph.13496 26096330

[14] Zahreddine H, Clubbe C, Baalbaki R, Ghalayini A, Talhouk SN. Status of native species in threatened Mediterranean habitats: the case of *Pancratium maritimum* L. (sea daffodil) in Lebanon. Biodivers Conserv. 2004; 120: 11–18.

[15] Dafni A, Cohen D, Noy-Mei I. Life-cycle variation in geophytes. Ann Mo Bot Gard. 1981; 68: 652–660.

[16] Arcangeli G. Sulla struttura e sulla disseminazione dei semi di *Pancratium maritimum*. Bull Soc Bot It. 1896; 8: 278–281.

[17] Werker E, Fahn A. Seed anatomy of *Pancratium* species from three different habitats. Bot Gaz. 1975; 136: 396–403.

[18] Eisikowitch D, Galil L. Effect of wind on the pollination of *Pancratium maritimum* L. (Amaryllidaceae) by hawkmoths (Lepidoptera: Sphingidae). J Anim Ecol. 1971; 40: 673–678.

[19] Medrano M, Guitián P, Guitián J. Breeding system and temporal variation in fecundity of *Pancratium maritimum* L. (Amaryllidaceae). Flora. 1999; 194: 13–19.

[20] Grassi F, Cazzaniga E, Minuto L, Peccenini S, Barberis G, Basso B. Evaluation of biodiversity and conservation strategies in *Pancratium maritimum* L. for the northern Tyrrhenian Sea. Biodivers Conserv. 2005; 14: 2159–2169.

[21] Sanaa A, Fadhel NB. Genetic diversity in mainland and island populations of the endangered *Pancratium maritimum* L. (Amaryllidaceae) in Tunisia. Sci Hortic. 2010; 125: 740–747.

[22] De Castro O, De Luca A, Menale B. Chloroplast inheritance in the sea daffodil (*Pancratium maritimum*, Amaryllidaceae) through controlled crosses, seed germination and molecular analyses. Plant Biosyst. 2016;

[23] De Castro O, Brullo S, Colombo P, Jury S, De Luca P, Di Maio A. Phylogenetic and biogeographical inferences for *Pancratium* (Amaryllidaceae), with an emphasis on the Mediterranean species based on plastid sequence data. Bot J Linn Soc. 2012; 170: 12–28.

[24] Medrano M, Guitián P, Guitián J. Patterns of fruit and seed set within inflorescences of *Pancratium maritimum* (Amaryllidaceae): non-uniform pollination, resource limitation or architectural effects? Am J Bot. 2000; 87: 493–501. 10766720

[25] Mira S. Strelles E, González-Benito ME. Seed longevity characteristics of Pancratium maritimum. Eurogard VI. 6th European Botanic Gardens Congress. May 28-June 2012 Chios Island, Greece, pp. 102–103.

[26] Keren A, Evenari M. Some ecological aspects of distribution and germination of *Pancratium maritimum* L. Israel J Bot. 1974; 23: 202–215.

[27] Balestri E, Cinelli F. Germination and early-seedling establishment capacity of *Pancratium maritimum* L. (Amaryllidaceae) on coastal dunes in the north-western Mediterranean. J Coastal Res. 2004; 20: 761–770.

[28] Curr RHF, Koh A, Edwards E, Williams AT, Davies P. Assessing anthropogenic impact on Mediterranean sand dunes from aerial digital photography. J Coast Conservat. 2000; 6: 15–22.

[29] Nikopoulos D, Nikopoulou D, Alexopoulos AA. Methods for the preservation of genetic material of *Pancratium maritimum* (Amaryllidaceae). J. Food Agric Environ. 2008; 6: 538–546.

[30] Schlacher TA, Schoeman DS, Dugan J, Lastra M, Jones A, Scapini F, McLachlan A. Sandy beach ecosystems: key features, sampling issues, management challenges and climate change impacts. Mar Ecol. 2008; 29(S1): 70–90.

[31] Demır Z, Müderrısoğlu H, Aksoy N, Aydin SO, Uzun S, Özkara H. Effects of second housing and recreational use on *Pancratium maritimum* L. population in western Black Sea region of Turkey. J Food Agric Environ. 2010; 8: 890–894.

[32] Prisco I, Carboni M, Acosta ATR. The Fate of Threatened Coastal Dune Habitats in Italy under Climate Change Scenarios. PLoS ONE. 2013; 8: e68850 10.1371/journal.pone.0068850 23874787 PMC3706318

[33] Ciccarelli D. Mediterranean coastal dune vegetation: Are disturbance and stress the key selective forces that drive the psammophilous succession? Estuar Coast Shelf Sci. 2015; 165: 247–253.

[34] Giovino A, Domina G, Bazanc G, Campisi P, Scibetta S. Taxonomy and conservation of *Pancratium maritimum* (Amaryllidaceae) and relatives in the Central Mediterranean. Acta Bot Gall. 2015; 162: 289–299.

[35] Ricciardi M, Nazzaro R, Caputo G, De Natale A, Vallariello G. La flora dell’isola di Ischia (Golfo di Napoli). Webbia. 2004; 59: 1–113.

[36] WCS & CIESIN. Last of the Wild Project, version 2, 2005 (LWP-2): global human footprint dataset (geographic) Global Human Influence Index (HII) Dataset (Geographic). NASA Socioeconomic Data and Applications Center (SEDAC), Palisades; 2005.

[37] R Development Core Team. R: A language and environment for statistical computing. R Foundation for Statistical Computing, Vienna; 2012.

[38] Di Maio A, De Castro O. Development and characterization of 21 microsatellite markers for *Pancratium maritimum* L. (Amaryllidaceae). Conserv Genet Resour. 2013; 5: 911–914.

[39] DeWoody J, Nason JD, Hipkins VD. Mitigating scoring errors in microsatellite data from wild populations. Mol Ecol Notes. 2006; 6: 95–57.

[40] Van Oosterhout C, Hutchinson WF, Wills DPM, Shipley P. MICRO-CHECKER: Software for identifying and correcting genotyping errors in microsatellite data. Mol Ecol Notes. 2004; 4: 535–538.

[41] Peakall R, Smouse PE. GenAlEx 6.5: Genetic analysis in Excel. Population genetic software for teaching and research-an update. Bioinformatics. 2012; 28: 2537–2539. 10.1093/bioinformatics/bts460 22820204 PMC3463245

[42] Chapuis MP, Estoup A. Microsatellite null alleles and estimation of population differentiation. Mol Biol Evol. 2007; 24: 621–631. 10.1093/molbev/msl191 17150975

[43] Rousset F. GENEPOP’007: a complete re-implementation of the GENEPOP software for Windows and Linux. Mol Ecol Resour. 2008; 8: 103–106. 10.1111/j.1471-8286.2007.01931.x 21585727

[44] Rice WR. Analysing tables of statistical tests. Evolution. 1989; 43: 223–225. 28568501 10.1111/j.1558-5646.1989.tb04220.x

[45] Gaetano J. Holm-Bonferroni sequential correction: An EXCEL calculator (1.2) (Microsoft Excel workbook). 2013; 10.13140/RG.2.1.4466.9927

[46] Goudet J. FSTAT version 2.9.3.*2* Department of Ecology and Evolution, Lausanne University, Lausanne; 2002.

[47] El Mousadik A, Petit R. High level of genetic differentiation for allelic richness among populations of the argan tree (*Argania spinosa* (L.) Skeels) endemic to Morocco. Theor Appl Genet. 1996; 92: 832–839. 10.1007/BF00221895 24166548

[48] Weir B, Cockerham C. Estimating F-statistics for the analysis of population structure. Evolution. 1984; 38: 1358–1370. 28563791 10.1111/j.1558-5646.1984.tb05657.x

[49] Rousset F. Genetic differentiation and estimation of gene flow from F-statistics under isolation by distance. Genetics. 1997; 145: 1219–1228. 9093870 10.1093/genetics/145.4.1219 PMC1207888

[50] Excoffier L, Laval G, Schneider S. Arlequin (version 3.0): an integrated software package for population genetics data analysis. Evol Bioinform Online. 2005; 1: 47–50. PMC2658868 19325852

[51] Pritchard JK, Stephens M, Donnelly P. Inference of population structure using multilocus genotype data. Genetics. 2000; 155: 945–959. 10835412 10.1093/genetics/155.2.945 PMC1461096

[52] Falush D, Stephens M, Pritchard K. Inference of population structure: extensions to linked loci and correlated allele frequencies. Genetics. 2003; 164: 1567–1587. 12930761 10.1093/genetics/164.4.1567 PMC1462648

[53] Falush D, Stephens M, Pritchard K. Inference of population structure using multilocus genotype data: dominant markers and null alleles. Mol Ecol Notes. 2007; 7: 574–578. 10.1111/j.1471-8286.2007.01758.x 18784791 PMC1974779

[54] Hubisz MJ, Falush D, Stephens M, Pritchard JK. Inferring weak population structure with the assistance of sample group information. Mol Ecol Resour. 2009; 9: 1322–1332. 10.1111/j.1755-0998.2009.02591.x 21564903 PMC3518025

[55] Evanno G, Regnaut S, Goudet J. Detecting the number of clusters of individuals using the software STRUCTURE: a simulation study. Mol Ecol. 2005; 14: 2611–2620. 10.1111/j.1365-294X.2005.02553.x 15969739

[56] Earl DA, von Holdt BM. STRUCTURE HARVESTER: a website and program for visualizing STRUCTURE output and implementing the Evanno method. Conserv Genet Resour. 2012; 4: 359–361.

[57] Jakobsson M, Rosenberg NA. CLUMPP: A cluster matching and permutation program for dealing with label switching and multimodality in analysis of population structure. Bioinformatics. 2007; 23: 1801–1806. 10.1093/bioinformatics/btm233 17485429

[58] Piry S, Luikart G, Cornuet JM. Bottleneck: a computer program for detecting recent reductions in the effective population size using allele frequency data. J Hered. 1999; 90: 502–503.

[59] Luikart G, Cornuet JM. Empirical evaluation of a test for identifying recently bottlenecked populations from allele frequency data. Conserv Biol. 1998; 12: 228–237.

[60] Cornuet J, Luikart G. Description and power analysis of two tests for detecting recent population bottlenecks from allele frequency data. Genetics. 1996; 144: 2001–2014. 8978083 10.1093/genetics/144.4.2001 PMC1207747

[61] Luikart G. Usefulness of molecular markers for detecting population bottlenecks and monitoring genetic change. Ph.D. Thesis. University of Montana, Missoula; 1997. 10.1046/j.1365-294x.1998.00414.x 9711862

[62] Luikart G, Allendorf FW, Cornuet J-M, Sherwin WB. Distortion of Allele Frequency Distributions Provides a Test for Recent Population Bottlenecks. J Hered. 1998; 89: 238–247. 9656466 10.1093/jhered/89.3.238

[63] Di Rienzo A, Peterson AC, Garza JC, Valdes AM, Slatkin M. Mutational processes of simple sequence repeat loci in human populations. Proc Natl Acad Sci. 1994; 91: 3166–3170. 8159720 10.1073/pnas.91.8.3166 PMC43536

[64] Strubbe D, Beauchard O, Matthysen E. Niche conservatism among non-native vertebrates in Europe and North America. Ecography. 2015; 38: 321–329.

[65] Olson DM, Dinerstein E, Wikramanayake ED, Burgess ND, Powell GVN, Underwood EC, et al Terrestrial Ecoregions of the World: a new map of life on Earth. BioScience. 2001; 51: 933.

[66] Barve N, Barve V, Jiménez-Valverde A, Lira-Noriega A, Maher SP, Townsen Peterson A, Soberóna J, Villalobos F. The crucial role of the accessible area in ecological niche modeling and species distribution modeling. Ecol Model. 2011; 222: 1810–1819.

[67] Di Febbraro M, Roscioni F, Frate L, Carranza ML, De Lisio L, De Rosa D, Marchetti M, Loy A. Long-term effects of traditional and conservation-oriented forest management on the distribution of vertebrates in Mediterranean forests: a hierarchical hybrid modelling approach. Divers Distrib. 2015; 21: 1141–1154.

[68] Hijmans RJ, Cameron SE, Parra JL, Jones PG, Jarvis A. Very high resolution interpolated climate surfaces for global land areas. Int J Climatol. 2005; 25: 1965–1978.

[69] Zuur AF, Ieno EN, Elphick CS. A protocol for data exploration to avoid common statistical problems. Methods Ecol Evol. 2010; 1: 3–14.

[70] Otto-Bliesner BL, Marshall SJ, Overpeck JT, Miller GH, Hu A. Simulating Arctic climate warmth and icefield retreat in the last interglaciation. Science. 2006; 311: 1751–1753. 10.1126/science.1120808 16556838

[71] Braconnot P, Otto-Bliesner B, Harrison S, Joussaume S, Peterchmitt J-Y, Abe-Ouchi A, et al Results of PMIP2 coupled simulations of the Mid-Holocene and Last Glacial Maximum–Part 1: experiments and large-scale features. Clim Past. 2007; 3: 261–277.

[72] IPCC. Summary for Policymakers In: Stocker TF, Qin D, Plattner G-K, Tignor M, Allen SK, Boschung J, et al, editors. Climate Change 2013: The Physical Science Basis. Contribution of Working Group I to the Fifth Assessment Report of the Intergovernmental Panel on Climate Change. Cambridge: Cambridge University Press; 2013.

[73] Thuiller W, Lafourcade B, Engler R, Araújo MB. BIOMOD—a platform for ensamble forecasting of species distribution. Ecography. 2009; 32: 369–373.

[74] Hanley JA, McNeil BJ. The meaning and use of the area under a receiver operating characteristic (ROC) curve. Radiology. 1982; 143: 29–36. 10.1148/radiology.143.1.7063747 7063747

[75] Allouche O, Tsoar A, Kadmon R. Assessing the accuracy of species distribution models: prevalence, kappa and the true skill statistic (TSS). J Appl Ecol. 2006; 43: 1223–1232.

[76] Landis JR, Koch G.G. The measurement of observer agreement for categorical data. Biometrics. 1977; 33: 159–174. 843571

[77] Swets JA. Measuring the accuracy of diagnostic systems. Science. 1988; 240: 1285–1293. 3287615 10.1126/science.3287615

[78] Marmion M, Parviainen M, Luoto M, Heikkinen RK, Thuiller W. Evaluation of consensus methods in predictive species distribution modelling. Divers Distrib. 2009; 15: 59–69.

[79] Fielding A, Bell J. A review of methods for the assessment of prediction errors in conservation presence/absence models. Environ Conserv. 1997; 24: 38–49.

[80] Liu C, Berry P, Dawson T, Pearson RG. Selecting thresholds of occurrence in the prediction of species distributions. Ecography. 2005; 3: 385–393.

[81] Algar AC, Kharouba HM, Young ER, Kerr JT. Predicting the future of species diversity: macroecological theory, climate change, and direct tests of alternative forecasting methods. Ecography. 2009; 32: 22–33.

[82] Buisson L. Thuiller W, Casajus N, Lek S, Grenouillet G. Uncertainty in ensemble forecasting of species distribution. Glob Chang Biol. 2010; 16: 1145–1157.

[83] Dubuis A, Pottier J, Rion V, Pellissier L, Theurillat J-P, Guisan A. Predicting spatial patterns of plant species richness: a comparison of direct macroecological and species stacking modelling approaches. Divers Distrib. 2011; 17: 1122–1131.

[84] Fitzpatrick MC, Sanders NJ, Ferrier S, Longino JT, Weiser MD, Dunn R. Forecasting the future of biodiversity: a test of single-and multi-species models for ants in North America. Ecography. 2011; 34: 836–847.

[85] Innangi M, D’Alessandro F, Fioretto A, Di Febbraro M. Modeling distribution of Mediterranean beech forests and soil carbon stock under climate change scenarios. Clim Res. 2015; 66: 25–36.

[86] Legendre P, Legendre LFJ. Numerical ecology. London: Elsevier; 2012.

[87] Wang IJ. Examining the full effects of landscape heterogeneity on spatial genetic variation: a multiple matrix regression approach for quantifying geographic and ecological isolation. Evolution. 2013; 67: 3403–3411. 10.1111/evo.12134 24299396

[88] Lesica P, Allendorf FW. When Are Peripheral Populations Valuable for Conservation? Conserv Biol. 1995; 9: 753–760.

[89] Abeli T, Gentili R, Mondoni A, Orsenigo S, Rossi G. Effects of marginality on plant population performance. J Biogeogr. 2014; 41: 239–249.

[90] Fascetti S, Potenza G, Castronuovo D, Candido V. Wild geophytes of ornamental interest in the native flora of southern Italy. Ital J Agron. 2014; 9: 99–106.

[91] Arafeh R, Kadereit JW. Long-distance seed dispersal, clone longevity and lack of phylogeographical structure in the European distributional range of the coastal *Calystegia soldanella* (L.) R. Br. (Convolvulaceae). J Biogeogr. 2006; 33: 1461–1469.

[92] Sanaa A, Abid SB, Boulila A, Messaoud C, Boussaid M, Fadhel NB. Modeling hydrochory effects on the Tunisian island populations of *Pancratium maritimum* L. using colored Petri nets. BioSystems. 2015; 129: 19–24. 10.1016/j.biosystems.2015.02.001 25659990

[93] Sanaa A, Abid SB, Boulila A, Messaoud C, Boussaid M, Fadhel NB. Ecological systems as computer networks: long distance sea dispersal as a communication medium between island plant populations. BioSystems. 2016; 144: 27–34. 10.1016/j.biosystems.2016.04.006 27060659

[94] Kadereit JW, Arafeh R, Somogyi G, Westberg E. Terrestrial growth and marine dispersal? Comparative phylogeography of five coastal plant species at a European scale. Taxon. 2005; 54: 861–876.

[95] Escudero M, Vargas P, Arens P, Ouborg NJ, Luceño M. The east-west-north colonization history of the Mediterranean and Europe by the coastal plant *Carex extensa* (Cyperaceae). Mol Ecol. 2010; 19: 352–370. 10.1111/j.1365-294X.2009.04449.x 20002603

[96] Corlett RT, Westcott DA. Will plant movements keep up with climate change? Trends Ecol Evol. 2013; 28: 482–488. 10.1016/j.tree.2013.04.003 23721732

[97] Lambeck K, Purcell A. Sea-level change in the Mediterranean Sea since the LGM: model predictions for tectonically stable areas. Quat Sci Rev. 2005; 24: 1969–1988.

[98] Lambeck K, Rouby H, Purcell A, Sun Y, Sambridg M. Sea level and global ice volumes from the Last Glacial Maximum to the Holocene. Proc Natl Acad Sci. 2014; 111: 15296–15303. 10.1073/pnas.1411762111 25313072 PMC4217469

[99] Correggiari A, Roveri M, Trincardi F. Late Pleistocene and Holocene evolution of the North Adriatic Sea. Il Quaternario. 1996; 9: 697–704.

[100] Vai GB, Cantelli L. Litho-Palaeoenvironmental maps of Italy during the last two climatic extremes two maps 1:1.000.000 In: Anotonioli F, Vai GB, editors. Explanatory notes. 32° IGC publications SELCA, Florence; 2004.

[101] Holdsworth M. The flowering of rain flowers. J W Afr Sci Ass. 1961; 7: 28–36.

[102] Burtt BL. The evolution and taxonomic significance of a subterranean ovary in certain Monocotyledons. Isr J Bot. 1970; 19: 77–90.

[103] Giorgi F, Lionello P. Climate change projections for the Mediterranean region. Glob Planet Chang. 2008; 63: 90–104.

[104] Shaltout M, Omstedt A. Recent sea surface temperature trends and future scenarios for the Mediterranean Sea. Oceanologia. 2014; 56: 411–443.

[105] Dickson ME, Walkden MJA, Hall JW. Systemic impacts of climate change on an eroding coastal region over the twenty-first century. Clim Chang. 2007; 84: 141–166.

[106] Rovere A, Furlani S, Benjamin J, Fontana A, Antonioli F. MEDFLOOD project: MEDiterranean sea-level change and projection for future FLOODing. Alp MedIterr Quat. 2012; 25: 3–5.

[107] Shaltout M, Tonbol K, Omstedt A. Sea-level change and projected future flooding along the Egyptian Mediterranean coast. Oceanologia 2015; 57: 293–307.

[108] Riahi K, Rao S, Krey V, Cho C, Chirkov V, Fischer G, Kindermann G, Nakicenovic N, Rafaj P. RCP 8.5—A scenario of comparatively high greenhouse gas emissions. Clim Chang. 2011; 109: 33–57.

[109] Van Vuuren DP, Stehfest E, den Elzen MGJ, Kram T, van Vliet J, Deetman S, et al RCP2.6: exploring the possibility to keep global mean temperature increase below 2°C. Clim Chang. 2011; 109: 95–116.

[110] European Environment Agency.What is the trend in mean sea level globally and across European seas? 2014. http://www.eea.europa.eu/data-and-maps/indicators/sea-level-rise-2/assessment (Accessed July 2016).

