# Expression of Concern: A Multi-Faceted Approach to Analyse the Effects of Environmental Variables on Geographic Range and Genetic Structure of a Perennial Psammophilous Geophyte: The Case of the Sea Daffodil *Pancratium maritimum* L. in the Mediterranean Basin

**DOI:** 10.1371/journal.pone.0268424

**Published:** 2022-05-10

**Authors:** The PLOS ONE Editors

After publication of this article [1], concerns were raised that collection of Sea Daffodil samples at the two Croatian sites required permits, contrary to what was stated in the article’s Ethics Statement. An official from Croatia advised PLOS that the species studied in this article is classified as Critically Endangered in Croatia and was strictly protected under the Nature Protection Act and related regulations during the time of the study.

Field collections for the study [1] were conducted between 2009–2013. The “Study species” Methods section of the *PLOS ONE* article [1] stated that the IUCN had not yet evaluated the species in question. According to the IUCN Red List [2], *Pancratium maritimum* was assessed in 2015 and the assessment was published in 2018, after the *PLOS ONE* article’s publication date. IUCN listed this species as an unthreatened species in the “least concern” (LC) category and noted that *Pancratium maritimum* is present in several protected areas (including in the Mediterranean) and has been legally protected in some regions as early as 2002 [2].

The Ethics statement in [1] stated in error that, “No specific permissions were required for the sampling in all locations.” The authors have reviewed local requirements in response to the concerns raised in this case and provided the information in S1 Table. This table reflects information and documentation available as of October 2021, but may not accurately represent the requirements or permits in place at the time of the collections (2009–2013). Unfortunately, documentation pertaining to several of the collection sites is no longer available due to the time that has passed and the passing of a collaborator (GV) who conducted the sampling in several locations.

In light of the limited information available at this time about the requirements that were in place at each collection site, and the unavailability of permits and/or related documentation, PLOS is unable to clarify whether the field collections reported in this article complied with all local requirements. Therefore, the *PLOS ONE* Editors issue this Expression of Concern.

## Supporting information

S1 Table Information received from the authors regarding local protections and requirements and the permits that were obtained for the field collections. (DOCX)
