# Supplementary material for: A Multi-Faceted Approach to Analyse the Effects of Environmental Variables on Geographic Range and Genetic Structure of a Perennial Psammophilous Geophyte: The Case of the Sea Daffodil Pancratium maritimum L. in the Mediterranean Basin
Source: PLoS One. 2016 Oct 17;11(10):e0164816. doi: 10.1371/journal.pone.0164816 (PMC5066950; doi:10.1371/journal.pone.0164816)
Supplement: S3 File — Graph of delta K values to determine the ideal number of groups that were present in the accessions of Pancratium maritimum and genetic clusters (K) as obtained for the 48 P. maritimum populations (867 individuals) using STRUCTURE (K = 4). (DOCX) [file pone.0164816.s003.docx]

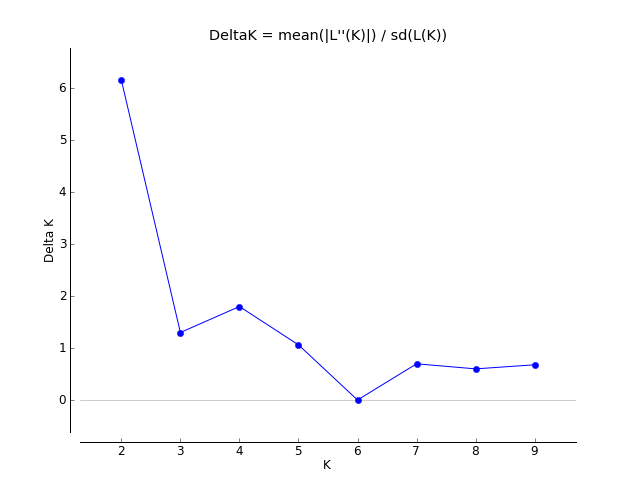


**Fig 1.** Graph of delta K values to determine the ideal number of groups present in the accessions of *Pancratium maritimum*, using six microsatellite loci and the Evanno method implemented in the STRUCTURE HARVESTER program.


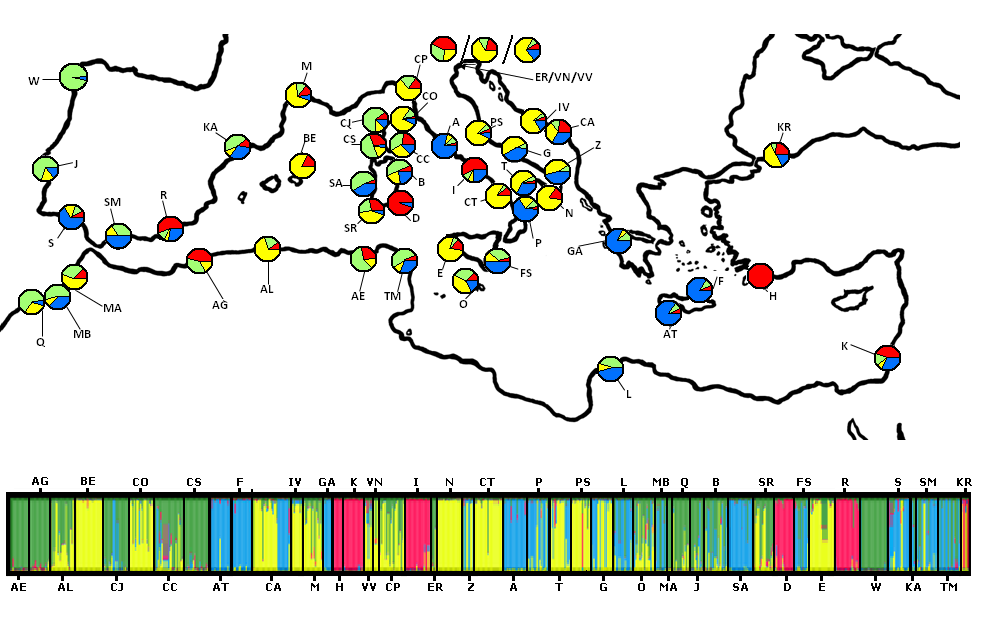


**Fig 2.** Genetic clusters (K) obtained for the 48 populations (867 individulas) of *Pancratium maritimum* populations using STRUCTURE (K = 4). Different colours indicate different genetic clusters.
