# Supplementary material for: Expression of Concern: A Multi-Faceted Approach to Analyse the Effects of Environmental Variables on Geographic Range and Genetic Structure of a Perennial Psammophilous Geophyte: The Case of the Sea Daffodil Pancratium maritimum L. in the Mediterranean Basin
Source: PLoS One. 2022 May 10;17(5):e0268424. doi: 10.1371/journal.pone.0268424 (PMC9089884; doi:10.1371/journal.pone.0268424)
Supplement: S1 Table — (DOCX) [file pone.0268424.s001.docx]

**S1 Table.** Information received from the authors regarding local protections and requirements and the permits that were obtained for the field collections.

| **Country** | **Is *Pancratium maritimum* protected in the country and/or region(s) where samples were collected?** | **Were permits obtained?** | **Permit records currently available?** |
| --- | --- | --- | --- |
| Algeria | No | No | - |
| Croatia | Yes | Unknown | No |
| France | No | No | Authors provided document of sampling from the Inventaire National du Patrimoine Naturel (INPN), France.  MNHN & OFB [Ed]. 2003-2022. Inventaire national du patrimoine naturel (INPN), https://inpn.mnhn.fr |
| Greece and Crete | Permit required from Ministry of Environment of Greece | Unknown | No |
| Israel | Yes | Yes | Yes |
| Italy | Regionally protected, including in three localities sampled | Yes, for one collection in a region (Toscana) where the species is not protected; unknown for other protected localities | Yes, for Toscana park collection  No for other collections |
| Libya | No protection law applies to the authors’ knowledge | No | - |
| Malta | Unclear | Unknown | No |
| Morocco | No protection law applies to the authors’ knowledge, nationally listed as Least Concern | No | - |
| Portugal | Currently considered as introduced species, further information needed regarding protection status | No | - |
| Spain | Regionally protected, including in two areas where samples were collected (Catologna, Baleari Island) | Unknown | No |
| Tunisia | No protection law applies to the authors’ knowledge, national red-listing currently in process | No | - |
| Turkey | Yes | Unknown | No |
